# Risk factors for candidemia: A case–control study

**DOI:** 10.1017/ash.2022.164

**Published:** 2022-05-16

**Authors:** Serin Edwin Erayil, Katelyn Tessier, Susan Kline

## Abstract

**Background:** *Candida* bloodstream infections (candidemia) have significant mortality and morbidity rates, as well as healthcare cost implications. Emerging multidrug-resistant *Candida* spp such as *Candida auris*, as well as increasing resistance among non–*albicans* species, which are becoming more prevalent, also raise concern. Understanding the epidemiology of this infection could enhance prevention and management efforts. We studied risk factors for candidemia. **Methods:** This matched case–control study was conducted at a university hospital from December 2019 through May 2021. Cases of candidemia were identified using positive blood-culture results. Controls were matched 5:1 to cases by age, sex, and month and year of admission. Risk factors of interest included total parenteral nutrition (TPN), central venous access (CVA), neutropenia, *Clostridium difficile*, pancreatic disease, *Candida* in urine culture, cancer, invasive procedures, H_2_ blockers, chemotherapy, antibiotic use, immunosuppression, and antifungal use. Bivariate conditional logistic regression models were used to study the association of individual factors with candidemia. Multivariable conditional logistic regression models were performed using factors with a P **Results:** Overall, 101 patients with candidemia and 505 matched controls were included. In the bivariate analysis, associations were detected between candidemia and TPN, CVA, pancreatic disease, invasive procedures, H_2_ blocker use, antibiotic use, and antifungal use (all Ps **Conclusions:** Associations of candidemia with recent antifungal use and pancreatic disease were relatively novel findings. Neutropenia was not an independent risk factor for candidemia in this study. Future directions include further evaluations of previous antifungal use in patients with candidemia to identify opportunities for possible intervention and antifungal stewardship.

**Funding:** None

**Disclosures:** None

